# Genetic Variations in *HSPA8* Gene Associated with Coronary Heart Disease Risk in a Chinese Population

**DOI:** 10.1371/journal.pone.0009684

**Published:** 2010-03-16

**Authors:** Meian He, Huan Guo, Xiaobo Yang, Li Zhou, Xiaomin Zhang, Longxian Cheng, Hesong Zeng, Frank B. Hu, Robert M. Tanguay, Tangchun Wu

**Affiliations:** 1 Department of Occupational and Environmental Health and the Ministry of Education Key Lab of Environment and Health, School of Public Health, Tongji Medical College, Huazhong University of Science and Technology, Wuhan, China; 2 Department of Cardiology, Union Hospital, Tongji Medical College, Huazhong University of Science and Technology, Wuhan, China; 3 Department of Cardiology, Tongji Hospital, Tongji Medical College, Huazhong University of Science and Technology, Wuhan, China; 4 Departments of Nutrition and Epidemiology, Harvard School of Public Health, Boston, Massachusetts, United States of America; 5 Laboratory of Cellular and Developmental Genetics, Department of Molecular Biology, Medical Biochemistry and Pathology, Faculty of Medicine, Université Laval, Québec, Canada; Innsbruck Medical University, Austria

## Abstract

**Background:**

There is ample evidence that Hsp70 takes part in the progress of coronary heart disease (CHD). This implies that genetic variants of Hsp70 genes such as *HSPA8* (HSC70) gene might contribute to the development of CHD. The present study aimed to investigate whether certain genetic variants of *HSPA8* gene are associated with CHD in Han Chinese people.

**Methodology/Principal Findings:**

A total of 2006 subjects (1003 CHD cases and 1003 age- and sex- matched healthy controls) were recruited. Genetic variants in the *HSPA8* gene were identified by sequencing of the gene in 60 unrelated Chinese. Four tag single nucleotide polymorphisms (tagSNPs) (rs2236659, rs2276077, rs10892958, and rs1461496) were selected and genotyped. The function of the significant SNP was evaluated using luciferase reporter assays in two cell lines. By sequencing the promoter and all exons and introns of the *HSPA8* gene, 23 genetic variants were identified. One promoter SNP rs2236659 was associated with susceptibility to CHD. Carriers of the “C” allele of rs2236659 had decreased CHD risk with odds ratio (OR) of 0.78 (95% CI: 0.62, 0.98; *P* = 0.033) after adjustment for conventional risk factors. Haplotype analyses indicated that haplotype *GCGC* contributed to a lower CHD risk (OR = 0.78, 95% CI: 0.65, 0.93; *P* = 0.006) compared with the common haplotype *AGGT*. In a transfection assay, the C allele of rs2236659 showed a 37–40% increase in luciferase expression of the reporter gene luciferase in endothelial and non-endothelial cells compared with the T allele.

**Conclusions/Significance:**

These findings suggest that genetic variants in *HSPA8* gene (especially promoter SNP rs2236659) contribute to the CHD susceptibility by affecting its expression level.

## Introduction

Coronary heart disease (CHD) is a complex disease with high morbidity and mortality. Very little is known about its genetic etiology. Heat shock protein 70 (HSP70), as a dominant chaperone in the HSPs families, can help in the assembly of newly synthesized proteins, in protein transport, and in the removal of damaged proteins [1]. In humans, the HSP70kDa family comprises 13 members, some of which show constitutive expression while others are stress inducible [2]. These isoforms have highly homogenous structure. They are all composed of a conserved ATPase domain, a peptide-binding domain, a middle region with protease sensitive sites, and a C-terminal domain[3], [4]. For instance, HSPA8, previously referred to as HSP73 or HSC70, shares 86% amino acid homology to inducible HSPA1A[3]. Consistent with their homogenous structure, these proteins have distinct but overlapping functions[3]. Thus both stress-inducible Hsp70 and constitutively expressed HSPA8 can perform some similar functions and are capable of protecting cardiac muscle cells against injuries like an oxidative challenge[5], [6]. There is much evidence indicating that Hsp70 can take part in the progress of CHD[7]–[9]. A previous study from our laboratory also demonstrated that genetic variants in the *HSPA1A* gene may be novel genetic risk markers for CHD[10]. Based on their high degree of structural homology and similar function in protecting against injuries in cardiac muscle cell, it is conceivable that the main constitutively-expressed member of the HSP70 family, HSPA8 might also be involved in the development of CHD and that single nucleotide polymorphisms (SNPs) and haplotypes of this gene may be associated with CHD and contribute to CHD susceptibility.

To test this hypothesis, we first sequenced and identified all SNPs in the *HSPA8* gene in 60 unrelated Han Chinese. We then selected 4 tagging SNPs (tagSNPs) to identify potential genetic markers of this gene for CHD susceptibility in a case-control study comprised of 1,003 CHD cases and 1,003 age- and sex- frequency matched controls in a Chinese population. We also examined the function of the SNPs associated with CHD susceptibility by performing a reporter gene luciferase activity assay in two types of cell lines.

## Results

### SNPs Identification in the *HSPA8* Gene in Han Chinese

In total we found 23 genetic variants in *HSPA8* gene with minor allele frequency (MAF) from 5.0% to 45.0% (**Table 1**). Among those, 6 novel SNPs had not been reported previously, including 301 c/*g* and 395 c/*g* (intron 1), 1255 t/c (intron 2), 1671 c/t (intron 3), 2692 gactc/– and 2716 *ca*/– (intron 5); The remaining 17 SNPs have been reported in NCBI as shown in **Table 1** (http://www.ncbi.nlm.nih.gov/genome). SNP rs11218942 (G > T> C) is triallelic and was not analyzed in further linkage disequilibrium analysis and tagSNPs selection.

**Table 1:** Variants discovered by sequencing all *HSPA8* exons and introns and 1 Kb upstream of transcript start site in 60 unrelated Chinese.

Variants	Position*	Gene pos.	Alleles	MAF (%)	Heterozygosity
rs11218942	−926	promoter	G > T> C	13.3	0.48
rs2236660	−703	promoter	T > C	14.2	0.24
rs2236659	−357	promoter	T > C	15.8	0.27
rs2236658	−308	promoter	A > G	35.8	0.46
rs2276077	8	5′ UTR	G > A	19.2	0.31
rs2276075	23	5′ UTR	C > T	19.2	0.31
rs1136141	48	5′ UTR	C > T	5.0	0.09
rs2276074	49	5′ UTR	C > G	30.0	0.42
New1†	301	intron1	C > G	19.2	0.31
New2 †	395	intron1	C > G	17.5	0.29
rs11218941	416	intron1	T > C	37.5	0.47
rs4935825	460	intron1	T > G	1.7	0.03
rs11823704	647	intron1	G > T	18.3	0.30
rs10892958	702	intron1	G > C	40.4	0.48
New3†	1255	intron2	T > C	20.0	0.32
New4†	1671	intron3	C > T	12.5	0.22
New5†	2692	intron5	Ins GACTC	12.5	0.22
New6†	2716	intron5	Ins CA	5.8	0.11
rs1461496	3201	intron6	C > T	44.5	0.49
rs1064585	3418	exon7	A > C	5.8	0.11
rs4936770	3950	intron8	A > G	45.0	0.49
rs3763897	4191	intron8	T > A	11.7	0.21
rs4802	4203	exon9	C > T	45.0	0.49

*Reference the transcription start code as +1.

† Novel SNP not reported in public database before.

### Selection of tagSNPs in *HSPA8* Gene

**Figure 1:**
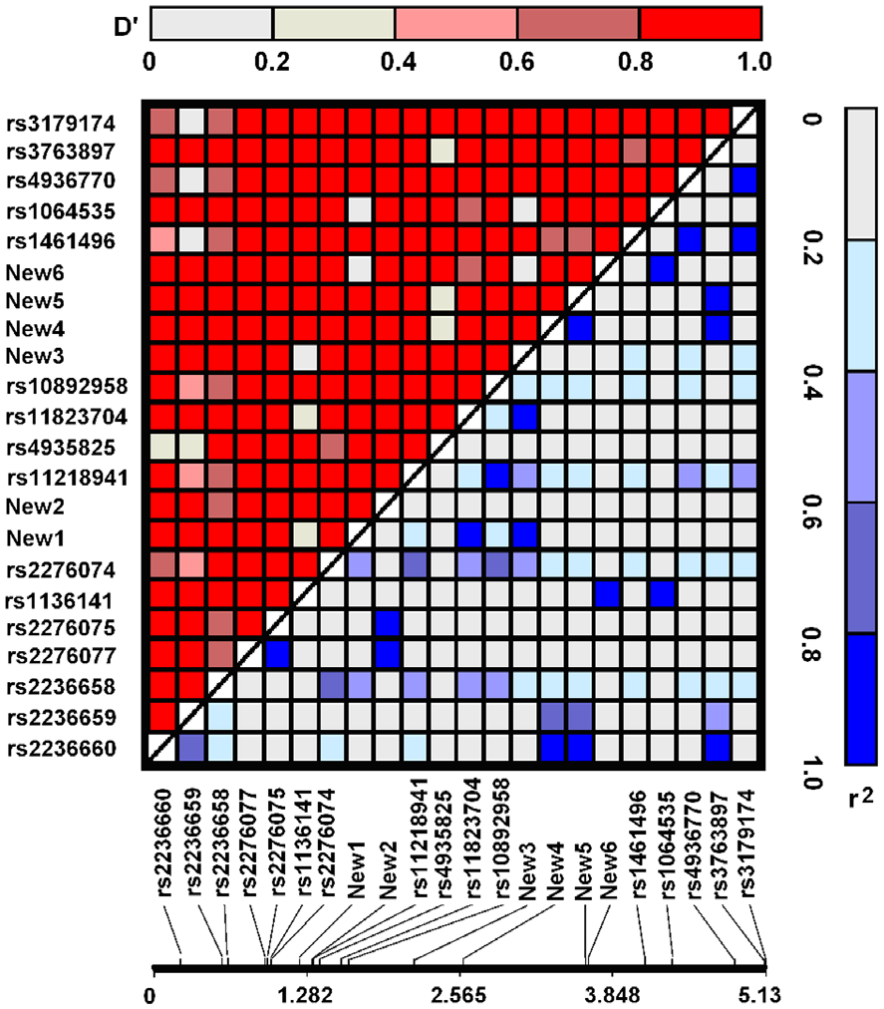
Linkage disequilibrium (D' and r^2^) between single nucleotide polymorphisms in *HSPA8* gene. Based on the data on the resequencing of the *HSPA8* gene, a total of 22 single nucleotide polymorphisms were analyzed by JLIN software to analyze linkage disequilibrium (D' and r^2^) between SNPs.

**Figure 2:**
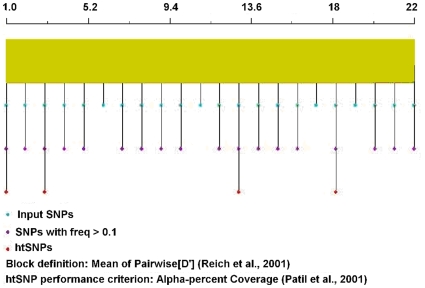
Construction of haplotype and tagging SNPs selection in *HSPA8* gene. A total of 22 single nucleotide polymorphisms (SNPs) were analyzed by htSNPer1.0 software to select tagSNPs. 1.0 indicated rs2236660 and 22.0, the last SNP rs4802. The results showed that rs2236660, rs2236658, rs10892958 and rs1461496 were selected for tagSNPs.

Based on the above sequencing data, linkage disequilibrium analysis results showed that all detected SNPs located in the same haploblock (**Figure 1**). The htSNPer1.0 software was used to pick out the tagSNPs[11], and finally four SNPs were selected as tagSNPs, including rs2236660, rs2236658, rs10892958 and rs1461496 (**Figure 2**). Because the sequences around the SNPs rs2236660 and rs2236658 are rich in GC and not suitable to be detected by TaqMan SNP allelic discrimination assay, we selected the other two SNPs rs2236659 and rs2276077, which are in high linkage with rs2236660 and rs2236658 for further analysis.

### General Characteristics of the Subjects

The general characteristics of the CHD cases and controls have been described in a previous study[12] and are summarized in **Table 2**. CHD patients had a higher prevalence of conventional vascular risk factors, including smoking, non-drinking, history of hypertension and diabetes mellitus, family history of CHD and higher level of FBG, whereas TC level in patients were surprisingly lower than in controls probably due to cholesterol-lowering treatment in the cases.

**Table 2:** General characteristics of CHD cases and controls*.

Variables	Cases (n = 1003)	Controls (n = 1003)	*P* Value
Sex (male/female)	638/365 (63.6/36.4)	637/366 (63.4/36.6)	0.94
Age (y)	59.7 (8.8)	59.5 (8.5)	0.31
Systolic blood pressure (mmHg)	134.6 (24.6)	131.6 (21.1)	0.004
Diastolic blood pressure (mmHg)	82.0 (14.4)	82.5 (11.1)	0.45
Body mass index	24.46 (3.51)	24.41 (3.37)	0.77
FBG (mmol/L)	6.66 (3.63)	5.32 (2.00)	<0.01
TC (mmol/L)	4.50 (1.05)	5.08 (1.30)	<0.01
TG (mmol/L)	1.73 (1.24)	1.69 (1.31)	0.45
Smoking (no/past or current)	435/564 (43.5/56.5)	535/468 (53.3/46.7)	<0.01
Smoking index (pack-year)			<0.01
0	435(43.5)	535(53.3)	
0–31	281(28.1)	273(27.2)	
31-	283(28.4)	195(19.5)	
Drinking (no/yes)	733/266 (73.4/26.6)	681/316 (68.3/31.7)	0.01
Past history			
Hypertension	705(70.4)	342(34.9)	<0.01
Diabetes mellitus	277(27.8)	63(6.5)	<0.01
Family history of cardiovascular disease	111(11.1)	31(3.1)	<0.01

*Data are means (standard deviation) for continuous variables and n (%) for categorical variables.

### *HSPA8* Genotypes and CHD Risk

The genotype frequencies of the four studied SNPs in *HSPA8* polymorphisms are summarized in **Table 3**. The distributions of SNPs rs2236659, rs2276077, rs10892958 and rs1461496 did not depart from the Hardy-Weinberg equilibrium in control group (*P* = 0.73, 0.62, 0.79 and 0.22 respectively). There was significant difference in genotype distribution of rs2236659 between CHD and controls. Adjustment for the conventional risk factors such as age, sex, pack-years of smoking, drinking, activity, hypertension, DM and family history of CHD did not appreciably alter the results. Compared with TT genotype of rs2236659, subjects with C allele had lower risk of CHD after adjustment for the conventional risk factors above (Crude odds ratio (OR) = 0.83, 95% CI: 0.69, 1.00; *P* = 0.049 and adjusted OR = 0.78, 95% confidence interval (CI): 0.62, 0.98; *P* = 0.033 respectively) (**Table 3**). Stratified analysis according to age (≤60 years and >60 years), sex and smoking status indicated that subjects with C allele of rs2236659 in men, older (>60 years) or smokers subgroups had significant lower risk of CHD. However in females, younger or non-smokers subgroups there were no significant differences. Further analysis indicated that there were no interactions between SNP rs2236659 and above factors respectively (data not shown). There were no significant differences between CHD and control group in SNPs of rs2276077, rs10892958 and rs1461496 before or after adjusting for conventional risk factors (*P* >0.05).

**Table 3:** Analysis of associations between the *HSPA8* polymorphisms and risk of CHD.

	Cases		Controls		Crude OR (95% CI)	Adjusted OR (95% CI)*
Genotype	N	(%)	N	(%)		
rs1461496						
GG	321	33.3	341	34.4	1.00	1.00
AG	435	45.1	464	46.9	1.00(0.82, 1.22)	1.01(0.79, 1.29)
AA	209	21.7	185	18.7	1.20(0.94, 1.54)	1.19(0.88, 1.62)
AG+AA	644	66.8	649	65.6	1.05(0.87,1.27)	1.06(0.84, 1.34)
rs10892958						
GG	336	34.6	305	30.7	1.00	1.00
GC	453	46.7	495	49.8	0.83(0.68, 1.02)	0.79(0.62, 1.02)
CC	181	18.7	194	19.5	0.85(0.66, 1.09)	0.82(0.60, 1.13)
GC+CC	634	65.4	689	69.3	0.84(0.69, 1.01)	0.80(0.64, 1.01)
rs2276077						
GG	721	74.6	749	75.5	1.00	1.00
AG	228	23.6	228	23.0	1.04(0.84, 1.28)	0.99(0.76, 1.28)
AA	18	1.9	15	1.5	1.25(0.62, 2.49)	2.00(0.81, 4.99)
AG+AA	246	25.5	243	24.5	1.05(0.86,1.29)	1.03(0.81,1.33)
rs2236659						
TT	652	68.0	624	63.7	1.00	1.00
CT	267	27.8	313	32.0	0.82(0.67, 0.99) †	0.76(0.59, 0.97) ‡
CC	40	4.2	42	4.3	0.91(0.58, 1.43)	0.93(0.54, 1.61)
CT+CC	307	32.0	355	36.3	0.83(0.69, 1.00) §	0.78(0.62, 0.98) ||

*Adjusted for age, sex, pack-years of smoking, drinking, activity, hypertension, diabetes milletus and family history of CHD.

†*P* = 0.043, ‡ *P* = 0.025, § *P* = 0.049, and ||*P* = 0.033 respectively when compared with TT genotype of rs2236659.

### Haplotype Associations with CHD Risk

All the pairwise LD measure D' of the four investigated tagSNPs in the *HSPA8* gene ranged from 0.90 to 0.96 (data not shown). A total of 14 and 12 haplotypes were estimated in the CHD and control groups respectively by using PHASE 2.0 software to reconstruct haplotypes based on the observed genotypes[13]. Among these, 5 haplotypes of *AGGT*, *GCGT*, *GCGC*, *GGAT* and *GGGT* were >1.0% (from left to right the order of polymorphic bases in haplotype is rs1461496, rs10892958, rs2276077, and rs2236659). The associations between the common haplotypes (covering 96.95% and 98.16% of allelic variance in CHD and controls, respectively) encompassing *HSPA8* polymorphisms and CHD risk were also examined. Compared with the highest-frequency haplotype of *AGGT*, the *GCGC* haplotype had 22% lower risk of CHD (OR = 0.78, 95% CI: 0.65, 0.93; *P* = 0.006). However, haplotype *GCGT*, which is only different from *GCGC* in rs2236659, had no significant difference compared with *AGGT* (OR = 1.00, 95% CI: 0.85, 1.17; *P* = 0.958), confirming the results of single SNP analyses that subjects with C allele of rs2236659 had lower risk of CHD (**Table 4**).

**Table 4:** Haplotype distribution of *HSPA8* in CHD and control group.

Haplotype *	CHD		Controls		OR (95% CI)	*P* value
	N	%	N	%		
*AGGT*	850	42.37	819	40.83	1.00	
*GCGT*	495	24.68	479	23.88	1.00(0.85, 1.17)	0.958
*GCGC*	317	15.80	392	19.54	0.78(0.65, 0.93)	0.006
*GGAT*	245	12.21	251	12.51	0.94(0.77, 1.15)	0.549
*GGGT*	38	1.89	28	1.40	1.31(0.80, 2.15)	0.291
Others †	61	3.04	37	1.84	1.59(1.04, 2.42)	0.031
Total	2006	100	2006	100	--	--

*Polymorphic bases were in 3′ to 5′ order and from left to right the order is rs1461496, rs10892958, rs2276077 and rs2236659.

† Other haplotype includes *ACGT*, *GGGC*, *AGGC*, *AGAT*, *GCAT*, *ACGC*, *AGAC*, *GGAC*, *ACAC*, and *ACAT* with frequency <1.0%.

### *HSPA8* Promoter Region Carrying the −357C (rs2226659) Leads to Higher Expression in a Reporter Assay

**Figure 3:**
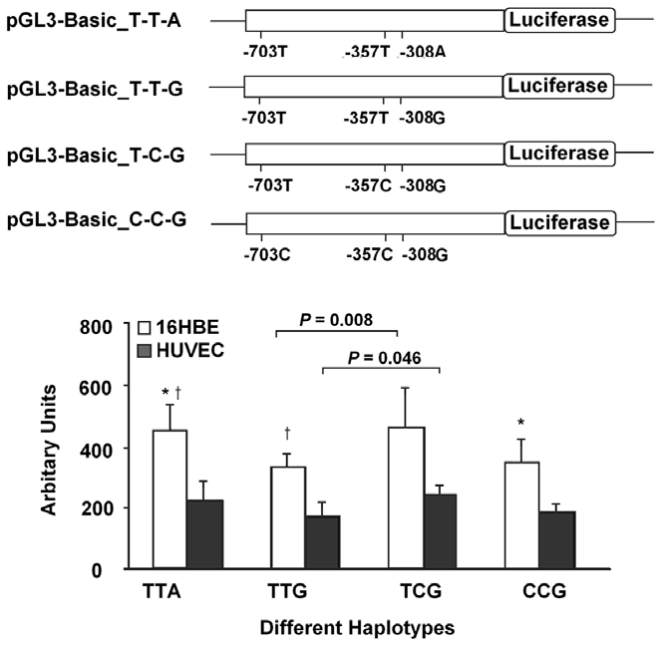
Effects of haplotypes in promoter of *HSPA8* on luciferase activity. Transient luciferase reporter gene expression assays with constructs containing different haplotypes of *HSPA8* promoter in human bronchial epithelial cells (HBE) and human umbilical vein endothelial cells (HUVEC). Upper: schematic representation of different reporter gene constructs. Variants of -703C/T, -357C/T, and -308A/G represent rs2236660, rs2236659, and rs2236658 respectively; Lower: luciferase expression of these constructs. All constructs were cotransfected with pRL-SV40 to standardize the transfection efficiency. Fold increase of luciferase activity was calculated by defining the activity of the empty pGL3-Basic vector as 1. All experiments were performed in triplicates at least in three independent transfection experiments and each value represents mean (standard deviation). *and † indicated that *P*<0.05, compared with each of the construct.

In order to understand the functional significance of the-357 T/C change, we used a reporter assay with luciferase. As shown in **Figure 3**, relative luciferase expression driven by the T-C-G containing promoter were 37–40% higher than that by the haplotype T-T-G containing promoter in the two types of cell lines (*P* = 0.008 for HBE and 0.046 for HUVEC). Because the two haplotypes of T-C-G and T-T-G are only different in -357T/C, these results suggested that the -357C variant (rs2236659) had a higher promoter activity than -357T allele. Similar results were obtained for the T-T-A haplotype when comparing with haplotypes T-T-G or C-C-G in HBE; however, there were no significant differences observed in HUVEC cells

## Discussion

Our study is the first one to examine the associations of variants of a constitutively expressed member of the HSP70 family, *HSPA8* and CHD susceptibility. Subjects with the C allele of rs2236659 had lower risk of CHD independent of other conventional risk factors. Further functional study in a cell reporter assay suggested that this association might be due to the increased promoter activity of the C allele of rs2236659 which may result in higher levels of expression of this HSPA8 protein.

HSPA8 is constitutively expressed and only mildly induced during stress situations[14]. It plays an important role in folding protein during their synthesis, transporting protein across membranes, regulating stress response and it is also involved in cell survival[4], [15]. In cardiac muscle cells, overexpression of HSPA8 attenuated oxidative injuries and enhanced cell survival[5]. This implies that HSPA8 might participate in the progress of CHD since it is believed that oxidative injuries are involved in the etiology of CHD[16]. Variants of *HSPA8* gene could affect HSPA8 levels and/or function. HSPA8 might take part in the development of CHD by two ways. First, as mentioned above it is believed that reactive oxygen species (ROS) are involved in the etiology of CHD[16]–[18]. This Hsp could protect against endogenous or exogenously generated ROS[5] and thus contribute to the progression of CHD. Second, this protein has also been reported to protect against hypoxia-induced apoptosis in hypoxia-induced apoptosis-resistant macrophages[19] and in the control of apoptosis during embryogenesis[20]. Other studies have found that HSPA8 protects cells against injuries by suppressing of apoptosis signaling and that its overexpression results in resistance against stress-induced caspase activation[4], [21]. Although to our knowledge no previous studies have investigated the role of HSPA8 in endothelium cells apoptosis, it might protect endothelium cells against apoptosis, which is believed to be the initiating event of atherogenesis and plays a crucial role in the transition from a stable endothelialized plaque to plaque erosion and thrombosis[22].

SNPs in the promoter of the *HSPA8* gene might affect its level of transcription and then lead to similar changes at the protein level. The SNP rs2236659 locates 357 bp upstream of transcriptional start code. In silico analysis using bioinformatics softwares of Alibaba2.0 (Niels Grabe, http://www.gene-regulation.com/pub/programs/alibaba2/) and TESS program (http://agave.humgen.upenn.edu/utess/tess), predicts that the C allele has stronger binding capacity with transcriptional factor sp1 when compared with the T allele. Consistent with the computation, our study confirmed that C allele of SNP rs2236659 leads to an increase in promoter activity and probably heightens synthesis level of the corresponding HSPA8 protein which could decreases the risk of CHD. By using global expression data available from human lymphoblastoid cell lines[23], we did not find any evidence that rs2236659 directly influences HSPA8 expression. However, further studies conducted in cardiac tissues, vascular smooth muscle and/or endothelial cells would be necessary to draw conclusions about the possible effects of the rs2236659 SNP on HSPA8 expression.

Several strengths of this study should be acknowledged. First, our population is racially homogeneous (all Han Chinese), which weakens the possible biases from population stratification. In addition, the findings from case-control association study were confirmed by detailed functional assays in both endothelial and nonendothelial cell lines strengthened the association of the *HSPA8* gene variations and CHD. However, three major limitations should also be mentioned. Firstly, like all case-control studies, selection bias (inclusion of patients surviving CHD) may exist and might influence interpretation of the results. Secondly, the controls selected without performing coronary angiography might include some false negative cases. However, because the prevalence of CHD in China is still low[24], and our controls all had normal ECG and no clinical symptoms before enrollment, the false negative cases in the controls are likely to be rare. Finally, replication is the best way to validate an association, however, the present matched case-control study is well designed and had enough power (>80%) to detect SNPs with risk ratios >1.35, 1.30, and 1.25, given an α of 0.05 and allele frequencies of 0.1, 0.3, and 0.5, respectively. In addition, our detailed functional assays conducted in both endothelial and nonendothelial cell lines confirmed and strengthened the associations of the *HSPA8* gene variations and CHD. However, these associations still need to be validated in other ethnic groups.

In summary, our case-control study and reporter assays results suggest that variants in *HSPA8* gene contribute to CHD susceptibility. Future studies are needed to validate these findings and further investigate potential mechanisms underlying the links between variations of *HSPA8* gene and CHD risk.

## Materials and Methods

### Screening for SNPs in *HSPA8* Gene

DNA samples extracted from whole blood of 60 randomly selected healthy Han Chinese (28 males and 32 females) were used to identify SNPs in *HSPA8* gene (GenBank accession NM_006597.3). Resequencing region included all *HSPA8* exons and introns and 1 Kb upstream of the transcript start site. Genomic DNA was amplified and then purified using the ethanol/NaAc method. The PCR products were used as templates for sequencing reactions with the BigDye Terminator kit v3.1 (Applied Biosystems, Foster City, CA, USA). Purified sequencing reactions were run on an ABI 3100 genetic analyzer. Sequence analysis, SNP detection, and genotype were performed using Sequencing Analysis 5.1.1 and DNAStar software. All primers and reaction conditions are displayed in **Table S1.**

### Human Subjects

The study design for this investigation has been described earlier [12]. Briefly, the study population was composed of 1,003 case patients and 1,003 age- (±5 years) and sex- frequency matched controls. All enrolled subjects were unrelated ethnic Han Chinese. CHD cases were enrolled from three hospitals (Tongji Hospital, Union Hospital, and Wugang Hospital) in Wuhan (Hubei, China) between May 2004 and October 2006. These cases were diagnosed according to WHO criteria or by coronary angiography (significant coronary artery stenoses ≥50% in at least one major coronary artery). Myocardial infarction was diagnosed by a representative set of ECG, cardiac enzyme values, and typical symptoms. Angina was defined as use of nitroglycerine, experience of typical chest pain, or ECG changes compatible with ischemic heart disease. In total, 1,078 patients diagnosed as having CHD were recruited; 1,003 of them (93.0%) consented to participate in the study and provided questionnaire information and blood samples. The control subjects resided in the same city as the cases and were judged to be free of CHD and peripheral atherosclerotic arterial disease by medical history, clinical examinations, and electrocardiography. The response rate for the controls was 92.4% (1,003 of 1,085). Sociodemographic information, past history, family history of cardiovascular disease, and lifestyle factors were obtained through questionnaire interview.

Subjects were classified as nonsmokers, former, or current smokers. Habitual physical activity was classified into four groups: little, light, moderate and vigorous. Subjects were considered to be hypertensive if their systolic blood pressure was ≥140 mmHg and/or diastolic pressure ≥90 mmHg or if they were already being treated with antihypertensive drugs. All subjects gave written consent after receiving a full explanation of the study. The Ethics Committee of Tongji Medical College approved this study.

### Genotyping of *HSPA8* Polymorphisms

Fasting venous blood was collected in 5 ml heparin tubes, and genomic DNA was isolated with a Puregene kit (Gentra Systems, Inc., Minneapolis, MN, USA). Genotyping was performed with TaqMan SNP allelic discrimination method on an ABI 7900HT real-time quantitative polymerase chain reaction (PCR) system (Applied Biosystems), in 384-well format. PCR reactions were carried out in reaction volume of 5 *µ*l containing 5 ng DNA, 2.5 *µ*l 2× TaqMan universal PCR Master Mix, No AmpErase UNG (Applied Biosystems), 0.125 *µ*l 40× Assay Mix (Applied Biosystems). PCR conditions included 95°C for 10 min followed by 40 cycles of 15 s at 92°C and 1 min at 60°C. Two blank controls (DNA hydration solution) and two replicate quality control samples were included in each 384-well format, and two replicate samples were genotyped with 100% concordance. The intensity of each SNP met the criteria of three clear clusters in two scales generated by SDS software (Applied Biosystems). The TaqMan primers and probes are displayed in **Table S2**. Finally, genotyping failed in 13 (1.30%), 9 (0.90%), 11 (1.10%) and 24 (2.40%) controls and 38 (3.80%), 33 (3.3 0%), 36(3.60%) and 44 (4.40%) cases in rs1461496, rs10892958, rs2276077 and rs2236659 locus respectively owing to DNA quantity or quality.

### Reporter Plasmids Construction

Because rs2236659 (-357T/C) was associated with CHD risk and located in the core promoter region of *HSPA8*, we evaluated whether this variant had allele-specific effect on its transcriptional activity. We constructed plasmid containing 3 promoter SNPs (rs2236660 [-703T/C], rs2236659 [-357T/C], and rs2236658 [-308A/G]). Firstly, we amplified the -1 to -780 promoter region of *the HSPA8* gene, and then inserted it into Kpn I/Hind III enzyme sites of pGL3-Basic (Promega, Madison, Wisconsin, USA). The first constructed plasmid contained the T-T-A haplotype (from left to right: -703T/C, 357T/C, and -308A/G) of *HSPA8* (**Figure 3**). Primer pairs of amplification and site-specific mutagenesis are listed in **Table S3**. The direction and sequence authenticity of the above constructs were validated by restriction analysis and direct sequencing.

### Transient Transfection and Luciferase Reporter Assays

Human umbilical vein endothelial cells (HUVEC) and human bronchial epithelial cells (HBE) with density of 6×10^4^ were seeded into 48-well plates and transfected with 100 ng pGL3-Basic plasmids or its constructs (defined as -703T/-357T/-308A, -703T/-357T/-308G, -703T/-357C/-308G, and -703C/-357C/-308G) and 1 ng pRL-SV40 (Promega) using Lipofectamine 2000 (Invitrogen, Carlsbad, California, USA) when grown to 70% confluence. Luciferase activity was measured at 24-hr using Dual-Luciferase Reporter Assay System (Promega) on a TD-20/20n luminometer (Turner Design, Promega). Each construct was tested in triplicates and the transfection experiments were performed three times independently. The results are denoted as relative luciferase activity (RLA) since the luciferase activity was normalized by Renilla luciferase activity and the empty pGL3-Basic vector.

### Biological Variables Determination

Fasting blood glucose (FBG), total cholesterol (TC), and triglyceride (TG) were assayed using standard laboratory procedures at the Department of Clinical Laboratory at the Wuhan Union Hospital.

### Statistical Analysis

A chi-square test was applied to compare categorical variables and the Hardy-Weinberg equilibrium of the polymorphisms. A multiple logistic regression analysis was used to evaluate the association between SNPs and CHD with appropriate adjustment of cardiovascular risk factors. The ANOVA test was used to examine the differences in luciferase reporter gene expression. The linkage relationship between the four SNPs in *HSPA8* gene was measured by the linkage disequilibrium (LD) coefficient (D') calculating by JLIN[25] and LDA program[26]. The htSNPer1.0 software was used to select tagSNPs in *HSPA*8gene[11]. All genotype data for each sample were taken to construct the haplotypes by using the PHASE 2.0 program[13]. *P*<0.05 was considered statistically significant. All data analyses were carried out using SPSS 12.0 software (SPSS Inc., Chicago, Illinois, USA). Power calculations were performed using Quanto 1.2.3 (available from http://hydra.usc.edu/gxe).

## Supporting Information

Table S1 Primers and PCR conditions for sequencing HSPA8 gene.
(0.06 MB DOC)

Table S2 Primers and probes for genotyping 4 TagSNPs in HSPA8 gene.
(0.03 MB DOC)

Table S3 Primer sequences used in amplification and reporter plasmids construction.
(0.03 MB DOC)
