# Supplementary material for: Genetic Variations in HSPA8 Gene Associated with Coronary Heart Disease Risk in a Chinese Population
Source: PLoS One. 2010 Mar 16;5(3):e9684. doi: 10.1371/journal.pone.0009684 (PMC2838785; doi:10.1371/journal.pone.0009684)
Supplement: Table S1 — Primers and PCR conditions for sequencing HSPA8 gene. (0.06 MB DOC) [file pone.0009684.s001.doc]

**Table S1 Primers and PCR conditions for sequencing *HSPA8* gene**

| **Primers** | **Primer Sequences**  **(5’-3’)** | **T_m_**  **(°C )** | **Annealing Temperature (°C )** | **Amplification**  **scope** | **Length of Fragment (bp)** |
| --- | --- | --- | --- | --- | --- |
| No1-forward | GCTTGGGCTAGTTGGGGA | 58.1 | 58.0 | -1069 | 470 |
| No1-reverse | ACCCACGTCCGAATCCTC | 57.3 |  | -600 |  |
| No2-forward | AACACGAAGGTGGGGCAT | 58.0 | 60.0 | -651 | 515 |
| No2-reverse | ACAAGACCCAATCACAAGC | 52.5 |  | -137 |  |
| No3-forward | GGTGAGTGCGTTATCGTGA | 54.5 | 60.0 | -230 | 456 |
| No3-reverse | CAAATACCGCTGCCATCC | 56.9 |  | 225 |  |
| No4-forward | GATGGCAGCGGTATTTGG | 56.9 | 58.5 | 209 | 480 |
| No4-reverse | CAAGAGGTAATAGTGCCCAT | 52.0 |  | 688 |  |
| No5-forward | GCTTAGACAGGAGGTGATGG | 54.4 | 56.6 | 653 | 539 |
| No5-reverse | GTTCAAACTTCAACCTCCTAC | 50.8 |  | 1191 |  |
| No6-forward | AAGTTTGAACCTTTGCTACC | 51.6 | 61.0 | 1182 | 524 |
| No6-reverse | ACTGGTTTCTGGTGTTGACA | 52.9 |  | 1705 |  |
| No7-forward | TGCCCAACAGTGCCCTAG | 57.2 | 59.4 | 1648 | 534 |
| No7-reverse | GACCTCAAAGATTCCATCCT | 52.9 |  | 2181 |  |
| No8-forward | TTGATGTGTCAATCCTCACTAT | 52.0 | 61.0 | 2138 | 578 |
| No8-reverse | AACAATCACTCATCACAGCG | 53.7 |  | 2715 |  |
| No9-forward | GTATCTCAATTAACCCTAAAGG | 50.7 | 62.0 | 2655 | 486 |
| No9-reverse | AATCCATCATCATAGCGAGT | 56.4 |  | 3139 |  |
| No10-forward | CTGATAACAGATAAAGGGAAGT | 50.5 | 56.5 | 3090 | 564 |
| No10-reverse | TGTCTTCCTTGCTCAAACGG | 58.6 |  | 3653 |  |
| No11-forward | TACACAGGTAATTTAAGGCTTT | 51.8 | 61.0 | 3586 | 552 |
| No11-reverse | CCTGACGCAATCTGATACTAAA | 55.3 |  | 4137 |  |
| No12-forward | TTGCGCCAGACTTTTAGTAT | 53.2 | 61.0 | 4104 | 537 |
| No12-reverse | AGACTCAAAGCAATTGTATGG | 52.3 |  | 4641 |  |
| No13-forward | AGTGCTCAGTTCGGGGTAAGAC | 60.1 | 61.0 | 1515 | 427 |
| No13-reverse | AACCGTTCAGTGTCCGTAAAG | 57.1 |  | 1941 |  |
| No14-forward | ATTACCCGTGCCCGATTT | 57.6 | 61.0 | 3413 | 585 |
| No14-reverse | GGAATGGTGGTATTACGCTTG | 57.5 |  | 3997 |  |
