# Supplementary material for: Genetic Variations in HSPA8 Gene Associated with Coronary Heart Disease Risk in a Chinese Population
Source: PLoS One. 2010 Mar 16;5(3):e9684. doi: 10.1371/journal.pone.0009684 (PMC2838785; doi:10.1371/journal.pone.0009684)
Supplement: Table S2 — Primers and probes for genotyping 4 TagSNPs in HSPA8 gene. (0.03 MB DOC) [file pone.0009684.s002.doc]

**Table S2 Primers and probes for genotyping 4 TagSNPs in *HSPA8* gene**

| **SNPs** | **Primers** | **Probes** |
| --- | --- | --- |
| rs2236659 | Forward primer：5’ CCTCCCGCGGAAGCT 3’ | VIC-5’ CATGCGTAGAGGTGGA-MGB 3’ |
| T/C | Reverse primer：5’GCGGGAGTCCTCAGTTACC 3’ | FAM-5’ ATGCGTAGAGGCGGA-MGB 3’ |
| rs2276077 | Forward primer：5’CGGAAACCGGTCTCATTGAACT 3’ | VIC-5’ CTCTTGGGTTTTTTG-MGB 3’ |
| A/G | Reverse primer：5’GGCCTGGCTCCAATAACGAA 3’ | FAM-5’ CTCTTGGATTTTTTG-MGB 3’ |
| rs10892958 | Forward primer：5’CAGGAGGTGATGGGCACTATTAC 3’ | VIC-5’ CCATACGTTTTTCC-MGB 3’ |
| C/G | Reverse primer：5’CCCTCATCCCTTAACAGAACACTT 3’ | FAM-5’ CCATACCTTTTTCC -MGB 3’ |
| rs1461496 | Forward primer：5’CTTATGTTGGCCAGTTCCTTCCT 3’ | VIC-5’ TCGCTCAAACATCCA-MGB 3’ |
| A/G | Reverse primer：5’GTGCTAGGGTCCTGCTAAGG 3’ | FAM-5’ CGCTCAGACATCCA -MGB 3’ |
