# Supplementary material for: Genetic Variations in HSPA8 Gene Associated with Coronary Heart Disease Risk in a Chinese Population
Source: PLoS One. 2010 Mar 16;5(3):e9684. doi: 10.1371/journal.pone.0009684 (PMC2838785; doi:10.1371/journal.pone.0009684)
Supplement: Table S3 — Primer sequences used in amplification and reporter plasmids construction. (0.03 MB DOC) [file pone.0009684.s003.doc]

**Table S3 Primer sequences used in amplification and reporter plasmids construction**

| **Purpose** |  | **Sequence** |
| --- | --- | --- |
| pGL3-Basic based constructs  (5’-*Kpn* I, 3’-*Hind* III) | -1 to -780 | 5’-ATCCGGTACCCTCCCTCCAATCCCAATCCTG-3’  5’-ATCCAAGCTTACCGGTTTCCGCCCGCCAC-3’ |
|  | T-T-A to T-T-G | 5’-CGGGGTCAGCCCTTGCCCAAAGCCACGCTAT-3’  5’-ATAGCGTGGCTTTGGGCAAGGGCTGACCCCG-3’ |
| Site-specific mutagenesis^*^ | T-T-G to T-C-G | 5’-GCGCATGCGTAGAGGCGGACGCTCCCCTCCC-3’  5’-GGGAGGGGAGCGTCCGCCTCTACGCATGCGC-3’ |
|  | T-C-G to C-C-G: | 5’-GACTCCCGCGCGCGGGCTCGCTGCGCCCCAC-3’  5’-GTGGGGCGCAGCGAGCCCGCGCGCGGGAGTC-3’ |

^*^ from left to right the order of constructs is rs2236660, rs2226659, and rs2236658.
